# Commentary: Efficacy and Safety of Transcranial Direct Current Stimulation as an Add-on Treatment for Bipolar Depression: A Randomized Clinical Trial

**DOI:** 10.3389/fnhum.2018.00480

**Published:** 2018-12-03

**Authors:** Zhen-Yu Hu, Xiaoli Liu, Hong Zheng, Dong-Sheng Zhou

**Affiliations:** Ningbo Kangning Hospital, Ningbo, China

**Keywords:** transcranial direct current stimulation (tDCS), bipolar depression, dorsolateral prefrontal cortex, Hamilton depression rating scale (HAMD), randomizad controlled trial

Bipolar disorder is a severe, recurrent psychiatric disorder, characterized by repeated remission and deterioration (Soares-Weiser et al., 2007). Bipolar disorder causes a high burden medical care for the individuals and society (Ferrari et al., 2016). Compared to manic episode, depressive one is much more frequent and prolonged in bipolar disorder patients (Suppes et al., 2005). Currently there are several therapeutic approaches for bipolar depression (BD), including pharmacological treatment, electroconvulsive therapy (ECT), cognitive behavioral therapy (CBT) and repetitive transcranial magnetic stimulation (rTMS) (Judd et al., 2002; Okumura and Ichikura, 2014; Blumberger et al., 2018).

Transcranial direct current stimulation (tDCS) is a non-invasive brain stimulation (NIBS) method with proven efficacy for neuropsychiatric disorders. It is also easily portable, with low cost and simple for manipulation. It delivers a weak current to the cortex through scalp electrodes and then induces changes in cortical excitability (Chang et al., 2015). Anodal stimulation facilitates depolarization of neurons, which enhances the cortex excitability, cathodal stimulation leads to hyperpolarization of neurons, which inhibits the cortex function (Nitsche et al., 2003; Hasan et al., 2011). tDCS stimulation leads to changes in connected cortical and subcortical regions as well (Stagg et al., 2010; Lang et al., 2015). Previous studies demonstrated the prospective potential of tDCS in unipolar depression (Shiozawa et al., 2014; Brunoni et al., 2016). However, few studies have been conducted on the treatment effectiveness of tDCS in BD.

Recently, Sampaio-Junior et al. conducted the first randomized sham-control clinical trial on 59 subjects, which intended to determine the efficacy and safety of tDCS for BD (Sampaio-Junior et al., 2018). Requirements for the participants are between the ages of 18 and 65, diagnosed BD, Hamilton Depression Rating Scale (HDRS-17) scored higher than 17, low suicide risk (evaluated by Mini-International Neuropsychiatric Interview questionnaire), in stable condition after 4-week pharmacologic treatment. The connections to the stimulator were concealed so that neither experimenter nor participant could determine the polarity of stimulation. HDRS-17, Montgomery-Åsberg Depression Rating Scale, Clinical Global Impression(CGI) depression scale, cumulative responses (defined as from baseline, HDRS-17 scores reduced >50% at week 2, 4, 6), remission rates were totally assessed at baseline, week 2, week 4, and week 6. In addition, rates of adverse events were recorded at week 2, 6.

Similar to many tDCS and rTMS studies, the clinical trial was administered to dorsolateral prefrontal cortex (DLPFC), which is a core region involves in cognitive control and emotion modulation. The location of DLPFC was determined by EASYstrap, a better positioning method compared to other methods. The anode (left) and cathode (right) electrode were placed on the bilateral DLPFC, respectively. 12 sessions of 2 mA (30 min) tDCS were performed, ten of which applied daily from Monday to Friday, with weekends off, two of which were performed at week 4 and 6 (study end point). The treatment schedule was same to previous studies, which made the results comparable (Brunoni et al., 2013).

The randomized sham-control trial continued for nearly 2 years. In active tDCS group, HDRS-17 score declined significantly when compared to the baseline. Through Kaplan-Meier analysis, the add-on intervention group shown to be more efficacious than sham group, showing higher cumulative response rate and remission rate. In addition, the change of Montgomery-Åsberg Depression Rating Scale indicated that active tDCS group had significant improvements than sham group (*P* < 0.05, but not significant in CGI). Besides skin redness, other adverse event rates were insignificantly different between active and sham group at each time point.

Previous studies only conducted small sample sized open-label protocol in mixed population of unipolar-bipolar subjects (Brunoni et al., 2012; Palm et al., 2012). The study was the first randomized double-blind sham-control trial to confirm the efficacy and safety of prefrontal tDCS applied to BD. The study would provide insights for further large multi-center studies. In addition, as we all know the BD patients suffered cognitive impairment (Wingo et al., 2009), it raises a concern whether tDCS treatment would enhance BD cognitive behaviors such as working memory, emotion process, attention bias and so on.

There are still some limitations in the present study. First, it is unknown if active tDCS was superior than sham in sustained remission. Perhaps this is due to the fact that remission analysis or tDCS design was not optimal? Second, CGI depression scale might not be sensitive for current sample population—given the less severity. Third, though utilizing proper randomization methods, the random distributions of baseline variables of the small sample were imbalanced. Forth, in integrity of blinding, as nearly three-fifths participants of each group identified correctly the allocation group, which might cause guinea pig effect.

In conclusion, the trial based on small sample presented the effectiveness, tolerance and safety of tDCS in bipolar depression patients. The promising result also inspires further research to demonstrate the efficacy of tDCS on BD in a large sample. Neuroscience studies indicated that individuals with BD displays cognitive deficits (Depp et al., 2016), so whether add-on tDCS intervention showing positive impacts on cognition requires more explorations.

The underlying neural mechanism of why tDCS can treat BD is yet unknown. Neuroplasticity as a neurophysiologic condition for depression is well recognized (Henn and Vollmayr, 2004), which is unclear if this is the case for BD patients. It is possible that tDCS induced neuroplasticity might contribute to alleviation of unipolar depression symptoms (Nitsche et al., 2009), which warrants further investigation.

## Author Contributions

D-SZ designed the study. Z-YH, XL performed and write the study. HZ contributed to revised work. All authors have read and approved the final version of the manuscript.

### Conflict of Interest Statement

The authors declare that the research was conducted in the absence of any commercial or financial relationships that could be construed as a potential conflict of interest.
